# Asociación entre el consumo de *Cannabis* y el riesgo de suicidio en adolescentes escolarizados de Santa Marta, Colombia

**DOI:** 10.7705/biomedica.4988

**Published:** 2020-06-30

**Authors:** Adalberto Campo-Arias, Yuli Paola Suárez-Colorado, Carmen Cecilia Caballero-Domínguez

**Affiliations:** 1 Programa de Medicina, Facultad de Ciencias de la Salud, Universidad del Magdalena, Santa Marta, Colombia Universidad del Magdalena Facultad de Ciencias de la Salud Universidad del Magdalena Santa Marta Colombia; 2 Programa de Psicología, Universidad Cooperativa de Colombia, Santa Marta, Colombia Universidad Cooperativa de Colombia Universidad Cooperativa de Colombia Santa Marta Colombia; 3 Programa de Psicología, Facultad de Ciencias de la Salud, Universidad del Magdalena, Santa Marta, Colombia Universidad del Magdalena Facultad de Ciencias de la Salud Universidad del Magdalena Santa Marta Colombia

**Keywords:** fumar marihuana, suicidio, adolescente, estudiantes, salud pública, estudios transversales, marijuana smoking, suicide, adolescent, students, public health, cross-sectional studies

## Abstract

**Introducción.:**

La prevalencia del consumo de *Cannabis* sigue en aumento en el mundo, especialmente, entre adolescentes. Dicho consumo se sobrepone al de cigarrillos y otras sustancias lícitas e ilícitas, y se ha asociado con síntomas depresivos e incremento del riesgo suicida. En el contexto colombiano poco se conoce sobre la relación entre el consumo de *Cannabis* y el riesgo de suicidio.

**Objetivo.:**

Evaluar la asociación entre el consumo de *Cannabis* y el riesgo suicida en adolescentes escolarizados de Santa Marta, Colombia.

**Materiales y métodos.:**

Se hizo un estudio transversal con una muestra probabilística de estudiantes de media vocacional entre los 13 y los 17 años de edad en colegios oficiales y privados. Se indagó sobre el consumo de *Cannabis* alguna vez en la vida y se cuantificó el riesgo suicida con la *Center for Epidemiologic Studies on Depression Scale*. Las puntuaciones superiores a ocho se categorizaron como riesgo suicida elevado. Se estimó la razón de oportunidad (OR) cruda y la ajustada.

**Resultados.:**

Participaron 1.462 estudiantes. La media para la edad fue de 14,4 años (desviación estándar, DE=0,8) y el 60,3 % correspondía a mujeres. La prevalencia del consumo de *Cannabis* alguna vez en la vida fue del 11,6 % (IC_95%_ 10,0-13,2) y el 13,3 % (IC_95%_ 11,6-15,0) de los estudiantes presentó riesgo suicida elevado. El consumo de *Cannabis* se asoció con dicho riesgo ajustado por otras variables (OR=1,88; IC_95%_ 1,23-2,88).

**Conclusiones.:**

El consumo de *Cannabis* se asoci**ó** con el riesgo suicida elevado en adolescentes escolarizados de Santa Marta, Colombia. Es necesario analizar otras variables que pueden mediar esta asociación.

## Abstract (en)

**Introduction:**

The use of *Cannabis* continues to increase worldwide, especially among adolescents. This use overlaps with cigarette smoking and other illicit and licit drugs and is associated with depressive symptoms and suicide risk. In our national context, little is known about the relationship between *Cannabis* use and suicide risk.

**Objective:**

To evaluate the association between *Cannabis* use and elevated suicide risk in high school adolescents in Santa Marta, Colombia.

**Materials and methods:**

We conducted a cross-sectional study with a probabilistic sample of high school students between 13 and 17 years of age from official and private schools. We analyzed the use of *Cannabis* some time in life and suicide risk was quantified using the suicide ideation scale designed by the Center for Epidemiological Studies on Depression. Scores greater than eight were categorized as a high suicide risk. The crude and adjusted opportunity ratio (OR) was estimated.

**Results:**

A total of 1,462 students participated. Their mean age was 14.4 years (SD=0.8) and 60.3% were women. The lifetime prevalence of *Cannabis* use was 11.6% (95%CI: 10.013.2) and 13.3% of the students reported high suicide risk (95%CI: 11.6-15.0). *Cannabis* consumption was associated with high suicide risk adjusted by other variables (OR=1.88; 95%CI: 1.23-2.88).

**Conclusions:**

*Cannabis* use was associated with high suicide risk in high school adolescents in Santa Marta, Colombia. Other variables should be studied in order to further examine this association.

En el contexto mundial el *Cannabis* es la sustancia ilegal más usada y la prevalencia anual de consumo equivale al 3,8 % de la población general ^1^. En la adolescencia, es frecuente comenzar el consumo de sustancias ilegales. En Colombia, aproximadamente el 10 % de los adolescentes reporta haber consumido *Cannabis* alguna vez en la vida ^2^. Dicho consumo se considera un comportamiento de riesgo para la salud y está relacionado con múltiples factores individuales y del contexto sociocultural. El consumo de *Cannabis* se asocia con consecuencias negativas para la salud física y mental a corto y a largo plazo ^3^.

Otras amenazas para la salud en la adolescencia son las lesiones accidentales y los problemas de salud mental. A nivel mundial, el suicidio es la segunda causa de muerte en adolescentes ^4^ y, en Colombia, la prevalencia actual de ideación suicida en adolescentes alcanza el 6,6 %, los planes suicidas, el 1,8 %, y la historia de intento de suicidio, el 2,5 % ^5^.

Los comportamientos suicidas representan un problema de salud pública, no solo por las implicaciones individuales, familiares, sociales y económicas, sino también por las dificultades para su prevención, dada la compleja imbricación de las variables asociadas con la observación clínica ^6^.

La asociación entre el consumo de *Cannabis* y el incremento del riesgo suicida no siempre ha sido constante en los estudios consultados. Por ejemplo, en una muestra de 86.256 adolescentes entre los 12 y los 15 años residentes en 21 países de bajos y medianos ingresos (Colombia no estuvo incluido), Carvalho, *et al.*^7^, observaron que el consumo de *Cannabis* alguna vez en la vida se asoció significativamente con el intento de suicidio (OR=2,30; IC_95%_ 1,74-3,04). Sin embargo, Chabrol, *et al*. ^8^, en su estudio de 972 estudiantes con un promedio de edad de 17 años, hallaron que el uso de *Cannabis* y la ideación suicida no se encontraban relacionados (beta=0,03; p=0,31). No obstante, en un par de revisiones sistemáticas y metaanálisis se concluyó que el consumo de *Cannabis*, con diferentes frecuencias o intensidad de uso, incrementaba en forma estadísticamente significativa el riesgo suicida ^9^^,^^10^.

Son varias las hipótesis que tratan de explicar la asociación entre el consumo de *Cannabis* y el riesgo suicida. El modelo racional o de bienestar plantea que el consumo de sustancias psicoactivas precipita o facilita los comportamientos suicidas en adolescentes cuando su uso trae consecuencias negativas ^11^^,^^12^. Otra propuesta propone que los adolescentes con comportamientos suicidas consumen sustancias psicoactivas como una forma de automedicación contra las emociones negativas o para manejar los síntomas de los episodios depresivos ^13^. Un tercer planteamiento propone que el consumo de *Cannabis* y los comportamientos suicidas se presentan porque existe una predisposición común, genética y ambiental ^14^.

En el presente estudio, se analizó la asociación entre el consumo de *Cannabis* alguna vez en la vida y el riesgo suicida ajustado por el funcionamiento familiar, y la presencia de síntomas depresivos y de estrés postraumático. En el contexto colombiano se conoce poco sobre la relación entre el consumo de *Cannabis* y el riesgo suicida y los hallazgos del presente estudio se suman al debate sobre los eventuales efectos de la legalización y del consumo medicinal del *Cannabis* desde la perspectiva de la salud mental pública ^12^^,^^15^^,^^16^.

El objetivo del estudio fue evaluar la asociación del consumo de *Cannabis* y el riesgo de suicidio en adolescentes escolarizados de décimo y undécimo grado de Santa Marta, Colombia.

## Materiales y métodos

Se presenta un análisis secundario de un estudio observacional analítico transversal que fue evaluado y aprobado por el Comité de Ética en Investigación de la Universidad del Magdalena, Santa Marta, Colombia. La participación en la investigación representó un riesgo mínimo para los estudiantes. Los padres o tutores de los participantes firmaron el consentimiento informado y los estudiantes dieron asentimiento voluntario. Se garantizó, asimismo, la confidencialidad y el anonimato de los cuadernillos de respuesta, acorde con la legislación colombiana ^17^.

Para el cálculo de la muestra, se consideró el universo de 10.810 estudiantes de media vocacional de décimo y undécimo grado de los registros de la Secretaría de Educación en el 2016.

Se estimó una muestra de 1.948 estudiantes y un 20 % de reposición ante eventuales pérdidas. La muestra se calculó para una prevalencia esperada del 50 %, lo que permitió contar con el mayor número de participantes. Se aceptó un error alfa del 5 % (precisión del 95 %) y un margen de error del 2 %. Este número total de participantes posibilitaba el cálculo de asociaciones con intervalos de confianza del 95 %, suficientemente estrechos para evitar un error de tipo II (beta), y hacer ajustes en las asociaciones hasta con un número de por lo menos 12 variables ^18^^,^^19^.

Los estudiantes se seleccionaron mediante muestreo probabilístico estratificado por conglomerados. Cada conglomerado correspondía a un salón de clase bajo el supuesto de que cada uno incluía 25 estudiantes. Los conglomerados se seleccionaron de manera aleatoria.

Los estudiantes entre 13 y 17 años diligenciaron, de forma anónima, un cuadernillo que incluía ítems del cuestionario sobre comportamiento de riesgo en jóvenes de la *Youth Risk Behavior Survey* (YRBS) para estudiantes de básica secundaria de los *Centers for Disease Control and Prevention* (CDC), cuya confiabilidad ha demostrado ser aceptable en diferentes poblaciones ^20^^-^^23^. El cuestionario también se ha empleado en adolescentes colombianos en estudios previos y los hallazgos han sido confiables ^24^.

Se recolectó la información sociodemográfica y se preguntó sobre el consumo de sustancias legales e ilegales (*Cannabis* y cocaína) alguna vez en la vida ^20^. Asimismo, se incluyeron varias escalas breves de medición para variables de interés, como la función familiar, los síntomas depresivos, los síntomas de estrés postraumático y la probabilidad de suicidio.

La función familiar se cuantificó mediante el cuestionario APGAR familiar, el cual se compone de cinco ítems que registran la percepción del sujeto sobre el funcionamiento del núcleo familiar durante los seis meses previos. Cada ítem ofrece cinco opciones de respuesta que se califican de cero a cuatro, por lo tanto, las puntuaciones totales pueden estar entre cero y veinte. Las puntuaciones menores de 15 se categorizaron como disfunción familiar ^25^. En adolescentes escolarizados de Colombia, esta escala presentó una aceptable coherencia interna, con un coeficiente alfa de Cronbach de 0,79 ^26^.

Los síntomas depresivos se cuantificaron con el cuestionario WHO-5 (*Well-Being Index*). Esta escala se compone de cinco ítems que exploran la presencia de síntomas durante las dos semanas anteriores. Ofrece cuatro opciones de respuesta para cada ítem, que se califican de cero a tres, en consecuencia, las puntuaciones totales pueden estar entre cero y 15. Las puntuaciones inferiores a nueve se categorizaron como riesgo de trastorno depresivo ^27^. Esta escala demostró un excelente desempeño psicométrico en adolescentes colombianos en un estudio anterior, con coeficiente alfa de Cronbach de 0,67 ^28^.

Se indagó sobre los síntomas de estrés postraumático mediante la escala breve de Davidson para trauma (*Davidson Trauma Scale*, DTS). Esta versión del instrumento se compone de cuatro ítems con cinco opciones de respuesta a las que se les asignan de cero a cuatro puntos. Las puntuaciones totales van de cero a 16. Las puntuaciones superiores a 12 se categorizaron como riesgo de estrés postraumático ^29^. En una muestra de participantes mayores de 15 años en Colombia, la versión extensa de este instrumento mostró una gran coherencia interna, con un coeficiente alfa de Cronbach de 0,97 ^30^.

El riesgo suicida se cuantificó con la escala del *Center for Epidemiologic Studies on Depression (CES-D), que* se compone de cuatro preguntas sobre manifestaciones suicidas durante la semana anterior. La escala brinda cuatro opciones de respuesta que se califican de cero a tres, o sea que las puntuaciones totales pueden estar entre cero y 12. Las puntuaciones superiores a ocho se categorizaron como riesgo suicida elevado ^31^. Este cuestionario ha tenido un buen rendimiento psicométrico en adolescentes escolarizados colombianos, con un coeficiente alfa de Cronbach de 0,86 ^32^.

### Análisis de datos

En el componente descriptivo se calcularon las distribuciones de frecuencia de las variables estudiadas, con intervalos de confianza de 95 % (IC_95%_) para las importantes. El consumo de *Cannabis* se consideró como la variable independiente, las variables demográficas como covariables y el riesgo suicida como la variable dependiente.

Para evaluar la asociación entre el consumo de *Cannabis* y el riesgo suicida, se determinó la razón de oportunidad (OR) con un IC de 95 %. El ajuste de la asociación por covariables y las variables de confusión se hizo mediante regresión logística. Para el proceso de ajuste, se siguieron las recomendaciones de Greenland, es decir que la variable mostrara valores de probabilidad menores de 0,20 en el análisis bivariado y que produjera una variación mayor del 10 % en la asociación entre consumo de *Cannabis* y el riesgo suicida ^33^.

Para garantizar la confiabilidad de las mediciones, se calculó la coherencia interna con el alfa de Cronbach ^34^ para todas las escalas que evaluaban el constructo en el presente estudio. El análisis se hizo en el programa IBM- SPSS™, versión 22 ^35^.

## Resultados

Un total de 1.547 estudiantes diligenciaron los cuadernillos, pero en 85 (5,5 %) de ellos se omitieron respuestas, por lo que se excluyeron del análisis y, en consecuencia, la muestra final fue de 1.462 estudiantes. La media de la edad fue de 14,4 años (desviación estándar, DE=0,8). La caracterización sociodemográfica de los estudiantes se presenta en el cuadro 1. Los instrumentos de medición mostraron una aceptable coherencia interna (cuadro 2).

**Cuadro 1:** Características sociodemográficas de los estudiantes

Variable	n	%
Edad (años)		
13-15	425	29,1
16-17	1.037	70,9
Sexo		
Femenino	882	60,3
Masculino	580	39,7
Grado		
Décimo	809	55,3
Undécimo	653	44,7
Colegio		
Oficial	1.123	76,8
Privado	339	23,2
Estrato		
I	396	27,1
II	329	22,5
III	410	28,0
IV	98	6,7
V	24	1,6
VI	15	1,0
Sin respuesta	190	13,0
Etnia/raza		
(reconocida por los propios participantes)		
Indígena	19	1,3
Negra (afrocolombiana)	152	10,3
Mulata	85	5,8
Blanca	389	23,9
Mestiza	785	53,7
Otra	55	3,8
Sin respuesta	18	1,2

**Cuadro 2:** Alfa de Cronbach de las escalas de medición

Instrumento	Alfa de Cronbach
Cuestionario APGAR familiar	0,82
Cuestionario WHO-5	0,82
Escala breve de Davidson para trauma	0,66
Escala de ideación suicida del *Center for* *Epidemiologic Studies on Depression Scale (CES-D*)	0,75

Del total de estudiantes, 170 (11,6 %) informaron haber consumido *Cannabis* alguna vez en la vida (IC_95%_ 10,0-13,2), 103 (7,0 %) estudiantes se categorizaron como en riesgo de trastorno depresivo durante las dos semanas anteriores (IC_95%_ 5,7-8,3), 1.172 (76,1 %) participantes presentaron disfunción familiar (IC_95%_ 73,9-78,3) y 194 (13,3 %), riesgo suicida elevado durante la semana anterior (IC_95%_ 11,6-15,0).

La asociación cruda entre haber consumido *Cannabis* alguna vez en la vida y el riesgo suicida elevado fue significativa (OR=2,37; IC_95%_ 1,60-3,49). Las covariables de edad, sexo y grado no modificaron la asociación entre el consumo de *Cannabis* y el riesgo suicida elevado. Las variables de disfunción familiar, riesgo de trastorno depresivo y riesgo de trastorno de estrés postraumático, se comportaron como variables de confusión y se consideraron para el ajuste de la asociación entre el consumo de cannabis y el riesgo suicida dado que cumplieron con los criterios de Greenland. Los modelos ajustados se presentan en el cuadro 3.

**Cuadro 3:** Modelo de ajuste de la asociación del consumo de *Cannabis* con el riesgo suicida elevado

Ajustado por	OR	IC_95%_
Modelo 1		
Disfunción familiar	1,88	1,23-2,88
Riesgo de trastorno depresivo		
Riesgo de estrés postraumático		
Modelo 2		
Disfunción familiar	1,97	1,31-2,98
Riesgo de trastorno depresivo		
Modelo 3		
Riesgo de trastorno depresivo	1,80	1,17-2,77
Riesgo de estrés postraumático		
Modelo 4		
Disfunción familiar	2,05	1,35-3,13
Riesgo de estrés postraumático		
Modelo 5		
Riesgo de estrés postraumático	2,15	1,42-3,24
Modelo 6		
Disfunción familiar	2,09	1,41-3,11
Modelo 7		
Riesgo de trastorno depresivo	2,19	1,46-3,29

## Discusión

En el presente estudio, se observó que el consumo de *Cannabis* alguna vez en la vida incrementó el riesgo de suicidio en adolescentes de media vocacional de Santa Marta, Colombia. Este hallazgo es coherente con investigaciones precedentes que evidenciaron que dicho consumo en adolescentes incrementaba la probabilidad de reportar la ideación, el plan y el intento de suicidio ^7^^,^^8^^,^^36^^-^^39^. No obstante, en el estudio de Chabrol, *et al.*, se reportó que el consumo de cannabis no se relacionó con la probabilidad de suicidio ^8^.

Estas disparidades en los hallazgos pueden explicarse por las diferencias en las características demográficas de la población de estudio, los múltiples factores asociados con el consumo de *Cannabis*^3^, las diferentes variables relacionadas con los comportamientos suicidas ^6^ y las distintas formas de medición de las variables analizadas, en particular, la cuantificación del riesgo suicida ^40^.

En la actualidad, se carece de la evidencia suficiente para explicar la relación entre el consumo de sustancias como el *Cannabis* y la probabilidad de suicidio. Como ya se anotó, hay varios modelos explicativos que permiten tomar medidas preventivas eficaces ^11^^-^^14^. No se deben minimizar los efectos negativos del consumo habitual y persistente de *Cannabis* sobre la salud mental, en especial, en adolescentes y adultos jóvenes ^10^^,^^12^^,^^15^^,^^16^. Es importante tener presente que la legalización del consumo de una sustancia psicoactiva no convierte su consumo en un comportamiento saludable.

Las investigaciones muestran que el consumo regular de sustancias legales, como el cigarrillo y el alcohol, representa un problema de salud pública, ya que incrementa en forma estadísticamente significativa la morbilidad y la mortalidad de los consumidores habituales ^4^.

El consumo ocasional de *Cannabis* suele producir efectos negativos menores, incluidas algunas alteraciones cognitivas mínimas que pueden incrementar el riesgo de accidentes debido al deterioro en la concentración y la coordinación motora. En ese sentido, el consumo ocasional de *Cannabis* propicia los ataques de pánico y los síntomas psicóticos, especialmente en aquellas personas que se inician en el consumo ^41^. Sin embargo, los datos disponibles sugieren que los efectos deletéreos del consumo de *Cannabis* guardan relación con la dosis o la magnitud de la exposición, es decir que las consecuencias negativas se observan con mayor probabilidad en consumidores habituales después de varios años de uso.

Clarke, *et al*. ^42^, en un estudio de casos y controles, observaron que el consumo de *Cannabis* durante la adolescencia temprana se asoció con el intento de suicidio en los primeros años de la adultez. Asimismo, Silins, *et al*. ^43^, señalaron que los adolescentes que iniciaban el consumo diario antes de los 17 años tenían un mayor riesgo de intento de suicidio (OR=6,83; IC_95%_ 2,04-22,90) y de deserción escolar (OR=2,70; IC_95%_ 1,55-5,00), menos logros académicos (OR=2,68; IC_95%_ 1,52-4,55) y mayor probabilidad de consumo de otras sustancias (OR=7,80; IC_95%_ 4,46-13,60). Por último, en una revisión sistemática y metaanálisis recientemente publicado, Gobbi, *et al*. ^10^, observaron que la fuerza de la relación con la ideación suicida era menor (OR=1,50; IC_95%_ 1,11-2,03) que con el intento suicida (OR=3,46; IC_95%_ 1,53-7,84), lo que sugiere que la relación es mayor a medida que se incrementa la gravedad del comportamiento suicida.

Este estudio contribuye a evidenciar la relación entre el consumo de *Cannabis* y la probabilidad de suicidio en una muestra probabilística de estudiantes de media vocacional de una ciudad colombiana con grandes desigualdades sociales y económicas. Las condiciones sociales y económicas negativas pueden predisponer al consumo de sustancias psicoactivas ilegales y a los comportamientos suicidas ^7^^,^^9^^,^^10^^,^^38^^,^^44^.

El estudio presenta, de todas maneras, la limitación propia de las investigaciones transversales que no permiten establecer sin equívoco la dirección de causalidad. Sin embargo, es poco probable que se cumpla el modelo racional en la asociación entre el consumo de *Cannabis* y el riesgo suicida. Es más probable que dicho consumo en adolescentes sea una forma de automedicación para afrontar las situaciones psicosociales negativas o adversas ^11^^-^^13^ y, en ese sentido, se necesitan estudios que superen esta limitación ^45^.

En conclusión, el consumo de *Cannabis* incrementó el riesgo suicida después de ajustar por la disfunción familiar y el riesgo de los trastornos de estrés postraumático y depresivo en adolescentes escolarizados de colegios públicos y privados de Santa Marta, Colombia. Deben estudiarse otras variables que pueden mediar esta asociación dadas las implicaciones del consumo de *Cannabis* en población adolescente para la salud pública y la tendencia mundial de legalización del consumo.
